# Datenstandards für Seltene Erkrankungen

**DOI:** 10.1007/s00103-022-03591-2

**Published:** 2022-09-23

**Authors:** Peter N. Robinson, Holm Graessner

**Affiliations:** 1 The Jackson Laboratory for Genomic Medicine, 10 Discovery Drive, 06032 Farmington, CT USA; 2 Institute for Systems Genomics, University of Connecticut, 06032 Farmington, CT USA; 3 Institut für Medizinische Genetik und Angewandte Genomik, Zentrum für Seltene Erkrankungen, Universitätsklinikum Tübingen, Tübingen, Deutschland

**Keywords:** Gesundheitsdatenstandard, Nosologie, Ontologie, Seltene Erkrankungen, Terminologie, Standards for healthcare data, Terminology, Ontology, Rare diseases, Nosology

## Abstract

Die Verwendung von einheitlichen Datenformaten (Datenstandards) im Gesundheitswesen dient vier Hauptzwecken: 1) dem Datenaustausch, 2) der Integration von Computersystemen und -instrumenten, 3) der Datenspeicherung und -archivierung und 4) der Unterstützung föderierter Datenbanken. Sie sind besonders wichtig für die Erforschung und die klinische Versorgung Seltener Erkrankungen (SE).

In dieser Übersicht stellen wir Standards im Gesundheitswesen vor und präsentieren eine Auswahl von Standards, die im Bereich der seltenen Krankheiten häufig verwendet werden. Die „Human Phenotype Ontology“ (HPO) ist der am häufigsten verwendete Standard zur Annotation phänotypischer Anomalien und zur Unterstützung der phänotypgesteuerten Analyse der diagnostischen Exom- und Genomsequenzierung. Es gibt zahlreiche Standards für Krankheiten, die unterschiedlichen Anforderungen entsprechen. Das „Online Mendelian Inheritance in Man“ (OMIM) und die „Orphanet Rare Disease Ontology“ (ORDO) sind die wichtigsten Standards, die speziell für seltene Krankheiten entwickelt wurden. Die „Mondo Disease Ontology“ (Mondo) ist eine neue Krankheitsontologie, die darauf abzielt, auf umfassende Weise Daten aus aktuellen Nosologien zu integrieren. Neue Standards und Schemata wie die „Medical Action Ontology“ (MAxO) und das „Phenopacket“ der Global Alliance for Genomics and Health (GA4GH) werden gegenwärtig eingeführt, um die Palette der verfügbaren Standards zur Unterstützung der Forschung an seltenen Krankheiten zu erweitern.

Um eine optimale Versorgung von Patienten mit SE in verschiedenen Bereichen des Gesundheitswesens zu ermöglichen, müssen die Standards für seltene Krankheiten besser in die elektronischen Ressourcen des Gesundheitswesens integriert werden, z. B. über den Standard „FHIR“ (Fast Healthcare Interoperability Resources).

## Abstract (en)

The use of standardized data formats (data standards) in healthcare supports four main goals: (1) exchange of data, (2) integration of computer systems and tools, (3) data storage and archiving, and (4) support of federated databases. Standards are especially important for rare-disease research and clinical care.

In this review, we introduce healthcare standards and present a selection of standards that are commonly used in the field of rare diseases. The Human Phenotype Ontology (HPO) is the most commonly used standard for annotating phenotypic abnormalities and supporting phenotype-driven analysis of diagnostic exome and genome sequencing. Numerous standards for diseases are available that support a range of needs. Online Mendelian Inheritance in Man (OMIM) and the Orphanet Rare Disease Ontology (ORDO) are the most important standards developed specifically for rare diseases. The Mondo Disease Ontology (Mondo) is a new disease ontology that aims to integrate data from a comprehensive range of current nosologies. New standards and schemas such as the Medical Action Ontology (MAxO) and the Global Alliance for Genomics and Health (GA4GH) phenopacket are being introduced to extend the scope of standards that support rare disease research.

In order to provide optimal care for patients with SE in different healthcare settings, it will be necessary to better integrate standards for rare disease with electronic healthcare resources such as the Fast Healthcare Interoperability Resources (FHIR) standard for healthcare data exchange.

## Hintergrund

Eine optimale Patientenversorgung, die alle beteiligten Gesundheitsdienstleister in einem zeitlichen Kontinuum und auf interdisziplinäre Art und Weise einbindet, setzt eine Zusammenarbeit der Akteure voraus. Der reibungslose Austausch von Gesundheitsdaten ist hierfür eine wichtige Voraussetzung. Er wird durch die Kommunikation verschiedener IT-Systeme ermöglicht.

Einheitliche Datenformate (Datenstandards) von Gesundheitsdaten sind dabei essentiell, um Patientendaten für eine genaue Diagnose oder einen genauen Behandlungsplan verfügbar zu machen, um Daten gemeinsam nutzen zu können, Prozesse zuverlässig wiederholen zu können, Vergleiche anzustellen und um Innovationen wie künstliche Intelligenz (KI) vorantreiben zu können. Digitale Systeme und Werkzeuge benötigen Standards und Normen, um beispielsweise die Qualität von Daten zu prüfen, aber auch um die Syntax und Semantik von Daten aus Quellen wie elektronischen Patientenakten und anderen digitalen Quellen prozessieren zu können. Aktuell verwendete allgemeine Gesundheitsdatenstandards sind in Tab. 1 aufgeführt.

**Table 1:** 

Standard	Medizinischer Bereich	Einsatzbereich des Standards (syntaktischer oder semantischer Standard)
*DICOM* (Digital Imaging and Communications in Medicine)	Bildgebung	Speicherung und Austausch von medizinischen Bilddaten und Metadaten (syntaktischer Standard)
*FHIR* (Fast Healthcare Interoperability Resources)	Patientenakten	Kommunikation der Systeme verschiedener Anbieter (syntaktischer Standard)
*ICD* (International Classification of Diseases)	Kodierung	Internationale statistische Klassifikation/Kodierung von Krankheiten und verwandten Gesundheitsproblemen (semantischer Standard)
*LOINC* (Logical Observation Identifiers Names and Codes)	Labormedizin	Verzeichnis allgemeingültiger Namen und Identifikatoren zur Bezeichnung und zum Austausch von Untersuchungsergebnissen aus dem Labor- und Vitaldatenumfeld (semantischer Standard)
*SNOMED CT* (Systematized Nomenclature of Medicine – Clinical Terms)	Medizinische Sachverhalte	Ontologiebasierter Terminologiestandard für die Medizin (semantischer Standard)

In Hinsicht auf die Bewertung der Notwendigkeit und der Wirkung von Gesundheitsdatenstandards ist DICOM^1^, der internationale Standard für die digitale Bildgebung und Kommunikation in der Medizin, ein sehr gutes Beispiel. Als einer der ersten überhaupt eingeführten Gesundheitsdatenstandards war und ist DICOM bei Bildarchivierung und Kommunikation sowie der Integration von medizinischen Bildgebungsgeräten verschiedener Hersteller äußerst erfolgreich. Die bereits mit der DICOM-Entwicklung verfolgten Ziele können als exemplarisch für die Zwecke aller Gesundheitsdatenstandards gelten [1].

Die 4 Hauptzwecke von Gesundheitsdatenstandards sind:

1. Datenaustausch,
2. Integration von Computersystemen und -instrumenten,
3. Datenspeicherung und Archivierung,
4. Unterstützung föderierter Datenbanken.

Das Ziel des Beitrags ist es, die wichtigsten Standards für die Erforschung Seltener Erkrankungen (SE)^2^ und für die klinische Versorgung betroffener Menschen vorzustellen. Im Folgenden werden zunächst die wichtigsten Gesundheitsdatenstandards für SE und deren Einsatzbereiche beschrieben. Anschließend werden speziell für die Humangenetik bzw. für die Versorgung von Menschen mit genetischen und anderen SEs entwickelte Standards vorgestellt, die von den „allgemeinen“ Standards lückenhaft abgedeckte Inhalte spezifizieren oder spezielle Algorithmen für die translationale Forschung oder zur Unterstützung der Differentialdiagnostik ermöglichen. Der Beitrag schließt mit einer Diskussion über Perspektiven einer verbesserten Integration von SE-Standards in die klinische Versorgung bzw. einer verstärkten Anbindung an die elektronische Patientenakte (ePA) ab.

## Datenstandards für Seltene Erkrankungen

Aufgrund der überwiegend genetischen Verursachung, der großen phänotypischen Heterogenität sowie der Seltenheit und geografischen Verteilung der SE-Patienten und -Experten sind Datenstandards insbesondere für die klinische Versorgung und die translationale Erforschung von SEs unerlässlich. Die Verwendungszwecke dieser Datenstandards korrespondieren dabei mit den oben genannten Hauptzwecken von Gesundheitsdatenstandards. Zu den spezifisch für SE wichtigen Standards gehören Standards für genetische und genomische Daten, ohne die der bemerkenswerte Erfolg der wissenschaftlichen Gemeinschaft bei der Aufklärung der molekularen Ursachen von Tausenden von monogenen Erkrankungen (Mendelian Disorders) in den letzten Jahrzehnten kaum möglich gewesen wäre. Standards wie der von der „Human Genome Variation Society“ (HGVS) für die Benennung von genetischen Varianten sowie das „Variant Call Format“ (VCF) zur Erfassung von in Exom- oder Genomsequenzierung identifizierten Varianten ermöglichen die effiziente und akkurate informatische Analyse von Varianten im diagnostischen sowie im Forschungsfeld als auch den Austausch von Daten über Varianten in Datenbanken wie „ClinVar“ [2]. In diesem Artikel werden wir uns jedoch weitgehend auf Standards für klinische Daten beschränken und verweisen den interessierten Leser auf die Übersicht in Tab. 2 und weitere Informationsquellen [3].

**Table 2:** 

Standard	Spezifischer Bereich	Einsatzbereich des Standards
*American College of Medical Genetics* (Variant Interpretation Guidelines)	Einschätzung der Pathogenität von genetischen Varianten	Regeln zur Einteilung von Sequenzvarianten in die 5 Klassen: 1) Normvariante („benign“), 2) wahrscheinliche Normvariante („likely benign“), 3) Variante unklarer klinischer Relevanz („variant of unknown significance“, VUS), 4) VUS, wahrscheinlich pathogen („likely pathogenic“) und 5) VUS pathogen („pathogenic“; [7])
GA4GH Phenopacket Schema (Global Alliance for Genomics and Health)	Schema zur Erfassung klinischer Informationen	Ein Datenschema der GA4GH, welches Informationen über phänotypische Abnormitäten, klinische Messwerte, Behandlungen, Biopsien und genetisch/genomische Befunde erfasst [13, 14]
*HGVS* (Human Genome Variation Society)	Benennung von genetischen Varianten	Syntax zur Beschreibung von verschiedenen Varianten auf DNA-, RNA- und Proteinebene [5]
*HPO* (Human Phenotype Ontology)	Beschreibung phänotypischer Abnormitäten	Ontologie zur Benennung von abnormalen klinischen Merkmalen (s. Text; [8, 9])
*MAxO* (Medical Action Ontology)	Klinisches Management	Die neue Medical Action Ontology beschreibt Behandlungen und andere medizinische Handlungen, welche dem klinischen Management dienen
*Mondo* (Mondo Disease Ontology)	Nosologie aller Erkrankungen	Eine Nosologie, die zahlreiche andere Nosologien miteinander verbindet und vereinheitlicht [12]
*OMIM* (Online Mendelian Inheritance in Man)	Monogene Erkrankungen	Zusammenfassung relevanter Informationen zu monogenen und ausgewählten anderen Erkrankungen bzw. zu Genen und deren Funktionen [4]
*ORDO* (Orphanet Rare Disease Ontology)	Nosologie Seltener Erkrankungen	Eine Nosologie (Krankheitsontologie) mit Informationen zu Erkrankungen und deren Prävalenzen, Genassoziationen und HPO-basierten Symptombeschreibungen aus der Orphanet-Ressource [10]
*ORPHAcodes*	ORPHAcodes	International geltendes Klassifizierungssystem für Diagnosen im Bereich der seltenen Krankheiten [11]
*VCF* (Variant Call Format)	Beschreibung von genetischen Varianten, die durch Next-Generation-Sequenzierung (NGS) nachgewiesen wurden	Schema zur Spezifizierung von genetischen Varianten, die durch NGS-Methoden wie Exom- oder Genomsequenzierung identifiziert werden; das VCF-Schema enthält verschiedene Felder mit Informationen über die Sequenzqualität und -tiefe und die Allelverteilung usw. [6]

### Datenstandards für klinische Daten Seltener Erkrankungen

Die 4 genannten Hauptzwecke von Gesundheitsdatenstandards sind auch für klinische Datenstandards relevant. Häufig verwendete klinische Standards beziehen sich auf phänotypische Merkmale und Erkrankungen sowie andere Themenfelder wie klinisches Management. In den folgenden Abschnitten werden die für klinische SE-Daten wichtigsten standardisierten Ontologien (Wissensdarstellungen), Nosologien (Krankheitsklassifikationen) und Schemata vorgestellt. Ontologien und Datenstandards spielen insbesondere bei den Seltenen Erkrankungen eine große Rolle, weil es auch Spezialisten schwerfällt, ohne informatische Werkzeuge alle für die optimale Patientenbetreuung erforderlichen Informationen zu finden.

#### Human Phenotype Ontology (HPO)

**Figure 1:**
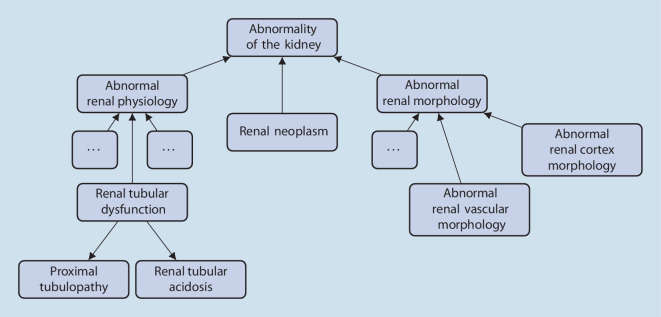

Die HPO ermöglicht eine umfängliche Erfassung von abnormen phänotypischen Merkmalen, die sich bei seltenen und nicht seltenen Erkrankungen manifestieren. Die sogenannten „Terms“ (Begriffe) der HPO entsprechen Symptomen, Befunden, auffälligen Ergebnissen von bildgebenden und anderen diagnostischen Verfahren und dergleichen mehr. Die Terms der HPO sind hierarchisch strukturiert, wobei die „Kinder“ eines Terms den spezifischen Formen eines übergeordneten „Eltern“-Terms entsprechen (Abb. 1).

Das HPO-Projekt umfasst nicht nur die Ontologie, sondern auch eine Bibliothek von Annotationen für über 8400 Seltene Erkrankungen. Die Ontologie bildet mit den Annotationen die informatische Grundlage für die Anwendung verschiedener Algorithmen, die zur Entscheidungsunterstützung oder bioinformatischen Analyse von genomischen Daten benutzt werden können. Zu den Anwendungsbereichen gehören Differentialdiagnostik, Forschung nach bislang unbekannten Krankheitsgenen, Systembiologie und Kohortenanalyse in elektronischen Krankenakten (EHR). Die HPO wurde 2008 an der Charité Berlin entwickelt [9] und ist seitdem zum De-facto-Standard in der internationalen humangenetischen Gemeinschaft geworden. Die HPO ist eine „anerkannte Ressource“ des International Rare Disease Research Consortium (IRDiRC; [15]) und wird von zahlreichen Projekten der Global Alliance for Genomics and Health (GA4GH) eingesetzt [14]. Die HPO ist in vielen nationalen Initiativen für genomische Medizin Standard, wie dem „100,000 Genomes Project“ von Genomics England, dem „National Institutes of Health (NIH) Undiagnosed Diseases Program and Network“, der „Deciphering Developmental Disorders Study“, und dem europäischen Projekt „Solve-RD – solving the unsolved rare diseases“ [15–18].

**Figure 2:**
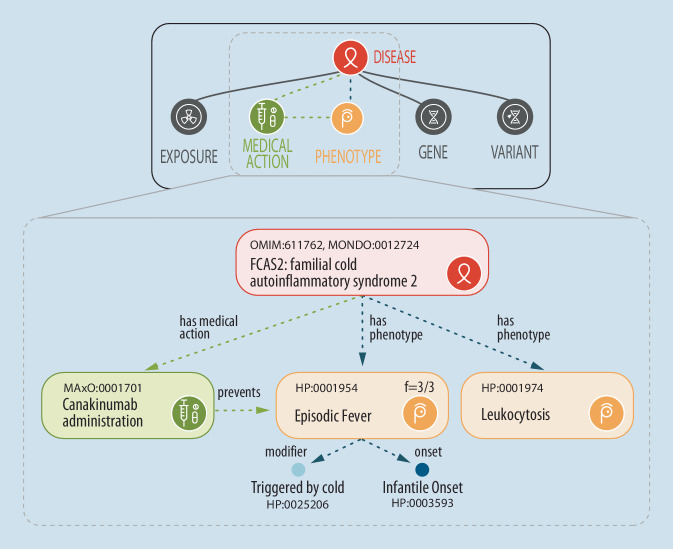

Ontologien wie die HPO können als Grundlage für Algorithmen zur informatischen Entscheidungsunterstützung dienen. Ontologien sind systematische Wissensrepräsentationen, die Informationen aus vielen heterogenen Quellen zu einem kohärenten Modell einer Domäne verbinden (Abb. 2; [19]). Die HPO ist die Grundlage für verschiedene Software-Tools wie „Exomiser“, welche zur Verbesserung der diagnostischen Ausbeute bei Initiativen wie dem „NIH Undiagnosed Diseases Program“ und dem „100,000 Genomes Project“ beigetragen haben [16, 20].

#### Nosologien für Seltene Erkrankungen

Verfügbare Krankheitsklassifikationen (Nosologien) unterscheiden sich in Strukturen, Formaten, Umfang und beabsichtigten Anwendungsbereichen. Die zwei am weitesten verbreiteten, für die Seltenen Erkrankungen spezialisierten Ressourcen, das „Online Mendelian Inheritance in Man“ (OMIM) und die „Orphanet Rare Disease Ontology“ (ORDO), haben unterschiedliche Schwerpunkte. ORDO ist eine Ontologie der seltenen monogenen und nicht-monogenen Erkrankungen mit Informationen zu Therapien, klinischen Merkmalen, ggf. Krankheitsgenen sowie Forschungsprojekten und Versorgungszentren. ORDO wurde 2014 veröffentlicht [10]. OMIM bietet keine Ontologie, sondern eine Terminologie (kontrollierte Liste) von Erkrankungen mit textuellen Informationen über Gene, Varianten, klinische Merkmale und ggf. Behandlungen. Traditionell werden monogene Erkrankungen vom OMIM-Team benannt, obwohl auch andere Benennungsregeln vorgeschlagen worden sind [21, 22].

Weitere Terminologien und Ontologien mit Krankheitsklassifikationen sind: „National Cancer Institute Thesaurus“ (NCIt), „SNOMED CT“, „Human Disease Ontology“, „ICD-10“, „MedGen“, spezialisierte Nosologien wie die „Sickle Cell Disease Ontology“ und dergleichen mehr [23–26]. Jede Ressource dient einem bestimmten Zweck und weist unterschiedliche Schwerpunkte sowie Stärken und Schwächen auf. Die „Mondo Disease Ontology“ wurde entwickelt, um diese Ressourcen so miteinander zu verbinden, dass mit unterschiedlichen Terminologien oder Ontologien annotierte klinische Daten interoperabel (austauschbar) sind [12].

#### Medical Action Ontology (MAxO)

MAxO ist eine Ontologie für medizinische Eingriffe, Therapien und andere dem klinischen Management dienliche Maßnahmen. Bei der Entwicklung von MAxO wurde der Schwerpunkt auf die Seltenen Erkrankungen gelegt, aber MAxO umfasst auch Terms für andere Erkrankungen. MAxO ist erhältlich über den „Ontology Lookup Service“ (OLS)^3^ des European Bioinformatics Institute (EMBL-EBI), eine erste Publikation wird derzeit vorbereitet. Vorgesehen ist, dass SEs auf der HPO-Webseite mit HPO-Terms sowie auch mit MAxO-Terms annotiert werden, um Artikel über Behandlungen für Seltene Erkrankungen leichter auffindbar zu machen. Künftig sollen auch Informationen des „Treatabolome-Projektes“ integriert werden. Das Treatabolome ist eine neue, vom europäischen Projekt „Solve-RD“ ausgehende Initiative zur Erstellung von systematischen Übersichten zum klinischen Management von Seltenen Erkrankungen [27, 28].

#### Das Phenopacket-Schema der GA4GH

Die 2013 gegründete Global Alliance for Genomics and Health (GA4GH) entwickelt eine Sammlung von aufeinander abgestimmten Standards für den Austausch von genomischen und klinischen Informationen [14]. Das Phenopacket-Schema ist ein Standard der GA4GH für klinische Informationen, wobei Informationen über eine Person und die bei ihr beobachteten phänotypischen Abnormitäten strukturiert erfasst werden, inklusive klinischer Messwerte, Krankheitsdiagnosen, ggf. Behandlungen und genetischer oder genomischer Untersuchungsbefunde. Ein Phenopacket beschreibt eine Person oder biologische Probe und ist gleichsam eine informatische Kasuistik. Es ermöglicht den informatischen Vergleich von phänotypischen Abnormitäten bei unterschiedlichen Patienten, was der Differentialdiagnose oder Kohortenbildung dienen kann [13]. Die Zertifizierung durch die „International Standards Organization“ (ISO 4454:2022) ist vor Kurzem erfolgt.

## Diskussion

Datenstandards sind für SEs von großer Wichtigkeit aufgrund der großen Anzahl der SEs, der Notwendigkeit der informatisch unterstützten Zusammenarbeit im Forschungs- und klinischen Versorgungskontext sowie aufgrund der dezentralen Erzeugung und Speicherung von SE-Daten. Aufgrund der sinkenden Kosten verschiedener Sequenzierungstechnologien ist zu erwarten, dass in den nächsten Jahren mehrere Millionen Patienten durch Genomsequenzierung untersucht werden. Damit verknüpft ist sowohl eine große Herausforderung für die Medizininformatik und Bioinformatik als auch die Möglichkeit, durch die geeignete Analyse großer Mengen an klinischen und genomischen Daten die molekularen Grundlagen monogener und komplexer Erkrankungen besser zu verstehen. Voraussetzung dafür ist, dass die Daten mit logisch aufeinander abgestimmten Standards kodiert werden.

Die ethischen und datenschutzrechtlichen Aspekte von Datenstandards und dem damit ermöglichten Datenaustausch sind nicht Gegenstand dieses Artikels. Wir möchten jedoch auf die Stellungnahme der GA4GH zum verantwortungsvollen Umgang mit klinischen Forschungsdaten verweisen [29].

Die Festlegung eines einheitlichen Datensatzes für die Erfassung von Daten über seltene Krankheiten in Europa ist ein sehr gutes Beispiel für die Anwendung und Sinnhaftigkeit der SE-Datenstandards. Primäres Ziel eines einheitlichen Datensatzes ist es, zu erreichen, dass die von den europäischen Registern erfassten Daten vergleichbar und die Datenregister interoperabel sind. Zugleich werden mit der Einführung des einheitlichen Datensatzes, der seinen Fokus auf Forschungsregister hat, auch Anforderungen für die Dokumentation von klinischen Primärdaten gemäß den SE-Datenstandards definiert. Damit werden die Voraussetzungen für epidemiologische, klinische oder pharmakologische Studien für SE geschaffen, die Primär- und Sekundärdaten aus ganz Europa gemäß einem einheitlichen und akzeptierten Standard sammeln und organisieren bzw. analysierbar machen können.

Der „Satz gemeinsamer Datenelemente für die Registrierung seltener Krankheiten“^4^ ist dabei das erste praktische Instrument, das von der „EU-Plattform für seltene Krankheiten“^5^ herausgegeben wurde, um die Interoperabilität der SE-Datenregister zu verbessern. Er definiert die Datenelemente, die von allen Registern für seltene Krankheiten in ganz Europa registriert werden müssen, und gibt Anweisungen, wie und in welchem Format jedes Datenelement registriert werden sollte. Die 16 definierten Datenelemente beziehen sich auf die persönlichen Daten der Patienten, die Diagnose, die Krankheitsgeschichte und den Behandlungsverlauf sowie auf Informationen, die zu Forschungszwecken bereitgestellt werden müssen. Für das Format der Mindestdatenelemente wird eine Reihe der oben aufgeführten Datenstandards verwendet, u. a. ORPHA-Codes, die HGVS-Nomenklatur, OMIM und ICF (Internationale Klassifikation der Funktionsfähigkeit und Behinderung).

Dieser Datensatz wird gegenwärtig durch den „Use Case CORD“ (Collaboration on Rare Diseases) der Medizininformatik-Initiative (MI-I) in der klinischen Dokumentation der Zentren für Seltene Erkrankungen implementiert, mit dem Ziel, diese Daten per Schnittstelle diversen Forschungsregistern zur Verfügung zu stellen.

Datenstandards, die für SE entwickelt worden sind, werden mehr und mehr auch für Erkrankungen verwendet, die nicht selten sind. Zum Beispiel werden HPO-Codes von mehreren Datenbanken für die „genomweite Assoziationsstudie“ (GWAS) verwendet [30, 31]. Die Präzisionsmedizin zielt auf Stratifizierung von Erkrankungen, um personalisierte Therapien zu ermöglichen. Die systematische Kodierung klinischer und genomischer Daten von Patienten mit komplexen Erkrankungen mit ggf. erweiterten Versionen der hier vorgestellten Standards könnte wesentlich dazu beitragen, verfügbare Daten optimal zu verwenden, um Präzisionsmedizin in der Zukunft für die Patientenversorgung am Point of Care informatisch anwendbar zu machen.

Die hier vorgestellten SE-Datenstandards wurden zunächst im Forschungskontext entwickelt, finden mit dem Eingang der Forschungsergebnisse in die klinische Versorgung der SE-Patienten aber auch Anwendung im klinischen Kontext. Vielleicht das wichtigste Beispiel ist die HPO, die, aus der humangenetisch-diagnostischen Forschung kommend, im aktuellen klinisch-genetischen Versorgungskontext ihren festen Platz gefunden hat. Der Folgeschritt, nämlich eine systematische Kodierung relevanter klinischer Daten in ePA, würde die Anwendung der informatischen Entscheidungshilfen in mehr klinischen Anwendungsfeldern erlauben, als heute möglich ist, was für die optimale Anwendung und Nutzung der genomischen Diagnostik in der klinischen Versorgung wesentlich ist. Deutschland hat für die Einführung der ePA erste Schritte getan. Arztbriefe, Befunde sowie Mutter- und Impfpass können damit am gleichen Ort gespeichert werden. Inwieweit klinische Datenstandards in der Zukunft in die ePA integriert werden, ist vor allem abhängig von den geltenden regulatorischen Bedingungen, die optimalerweise in einer begleitenden gesellschaftlichen Diskussion konsentiert werden sollten.

## Fazit und Ausblick

In dieser Übersicht haben wir einige der wichtigsten SE-Datenstandards vorgestellt. Diese Standards sind schon heute aus dem klinischen Alltag insbesondere des Humangenetikers nicht mehr wegzudenken und werden mit der wachsenden Bedeutung der genomischen Medizin an Bedeutung gewinnen. In der Zukunft sollten die verbesserte Integration von SE-Standards in die ePA und damit verbundene Standards wie FHIR (Fast Healthcare Interoperability Resources, ausgesprochen wie das englische Wort „fire“) im Fokus stehen, um Informationen und Wissen über SEs sowie entsprechende informatische Werkzeuge einem breiteren medizinischen Anwendungskreis zugänglich zu machen.

## Footnotes

1 Vgl. https://www.dicomstandard.org/.

2 Als „Seltene Erkrankungen“ (SE) gelten in der Europäischen Union (EU) Krankheiten, von denen nicht mehr als 5 von 10.000 Menschen betroffen sind.

3 http://www.ebi.ac.uk/ols.

4 https://eu-rd-platform.jrc.ec.europa.eu/set-ofcommon-data-elements_en.

5 https://eu-rd-platform.jrc.ec.europa.eu/_en.
